# Pathologically confirmed brain metastases from primary uterine cervical tumors: two cases and a literature review

**DOI:** 10.1186/s12957-019-1720-7

**Published:** 2019-10-31

**Authors:** Yalan Bi, Lei Li

**Affiliations:** 1 Department of Pathology, Peking Union Medical College Hospital, Peking Union Medical College & Chinese Academy of Medical Science, Beijing, 100730 China; 2 Department of Obstetrics and Gynecology, Peking Union Medical College Hospital, Peking Union Medical College & Chinese Academy of Medical Science, Shuaifuyuan No. 1, Dongcheng District, Beijing, 100730 China

**Keywords:** Cervical cancer, Brain metastasis, Pathogenesis, Chemotherapy, Radiotherapy

## Abstract

**Background:**

Pathologically confirmed brain metastasis from primary cervical cancer is extremely rare. Herein, we report two cases of intracranial metastasis from cervical cancer that were histopathologically confirmed after surgical excision. In addition, we conducted a literature review to characterize the clinical manifestation, pathogenesis, and treatment of these patients.

**Case summary:**

Among the 1800 patients with primary cervical cancer who received therapy at our center from 2010 to 2018, two patients (0.1%) had definite histopathological evidence of brain metastasis. A 46-year-old female who had a history of poorly differentiated stage IIB cervical cancer with neuroendocrine differentiation presented with a solitary mass in the right occipital lobe 26 months after the initial diagnosis. She underwent surgery and chemotherapy but died of disease progression 9 months later. Another 55-year-old female diagnosed with poorly differentiated stage IVB cervical squamous cancer presented with a solitary mass in the right frontal lobe 16 months after simple hysterectomy. Twelve months later, multiple lesions were observed in the bilateral frontal-parietal lobe. The lesions were treated by surgery and stereotactic radiosurgery. The patient died of multiple organ failure 14 months later.

**Conclusion:**

The pathogenesis and best management of brain metastasis from cervical cancer are not clear. Highly invasive subtypes or advanced cancer stages may be the key clinicopathological factors of brain metastasis. Surgical treatment is warranted in patients with a good health status and without metastasis to other sites.

## Introduction

Cervical cancer (CC) is the fourth most frequently diagnosed cancer and the fourth leading cause of cancer-related death in females, with an estimated 570,000 cases and 311,000 deaths worldwide in 2018 [1]. While a typical treatment consisting of a combination of surgery, radiotherapy, and chemotherapy has been established for early-stage or locally advanced CC, no standard treatment for metastatic disease has yet been established [2]. The 5-year survival rate in localized CC is 91.5%; however, it is merely 16.5% in cases of metastasis [3]. CC mainly spreads locally to the pelvic and para-aortic lymph nodes via the lymphatic system [4]. However, CC can also metastasize via the hematogenous pathway to more distant organs, with common sites being the lungs, liver, and bones [5]. Brain metastasis from primary CC is very rare, occurring in 0.4 to 2.3% of all patients [6]. To date, only approximately 140 cases of brain metastasis from CC have been reported [7]. Even fewer cases have been pathologically confirmed. Notably, an increase in brain metastasis has recently been observed [8]. Due to its rarity, no prospective clinical trials have been conducted to investigate optimal treatment strategies and prognostic factors, and poor overall survival—estimated to range from 2 to 8 months after the diagnosis of brain metastasis—has been reported [9].

In this report, we describe two cases of primary CC metastasis to the brain and conducted a literature review to investigate the clinical presentation, treatment, and prognosis of these patients.

## Case presentation

From January 2010 to December 2018, a total of 1800 patients with primary CC received therapy in our center, and recurrence occurred in 140 patients. After reviewing the medical cases, four patients (0.2%) had suspected brain metastasis, and metastasis was confirmed in two patients (0.1%) by pathological evaluation.

### Case 1

A 46-year-old woman (gravidity 1, parity 1) was referred to the study center in May 2015 after she complained of irregular vaginal bleeding for 2 years. A pelvic examination revealed a 5-cm cervical tumor involving the upper third of the vagina and infiltrating the distal compartment of the parametrium. A pelvic magnetic resonance imaging (MRI) scan revealed a 5.3 × 4.8 × 4.0 cm pelvic mass. Computed tomography (CT) scans of the chest and abdomen were negative for metastatic disease. Cervical biopsy confirmed poorly differentiated cervical carcinoma graded as International Federation of Gynecology and Obstetrics (FIGO) stage IIB. Immunohistochemical staining was positive for CK7, CgA, ER, PR, and PAX-8 expression but negative for CEA, Napsin A, P16, P40, P63, Syn, TTF-1, and CD56 (NK1) expression. The Ki-67 index was 70%. Primary treatment consisted of concurrent chemoradiotherapy: external radiation to the pelvis (60.0 Gy/28 f) followed by intracavitary brachytherapy (30 Gy/5 f) and concurrent intravenous cisplatin (40 mg/m^2^/week) for 5 weeks. Complete response was achieved, as determined by imaging evaluation.

**Fig. 1:**
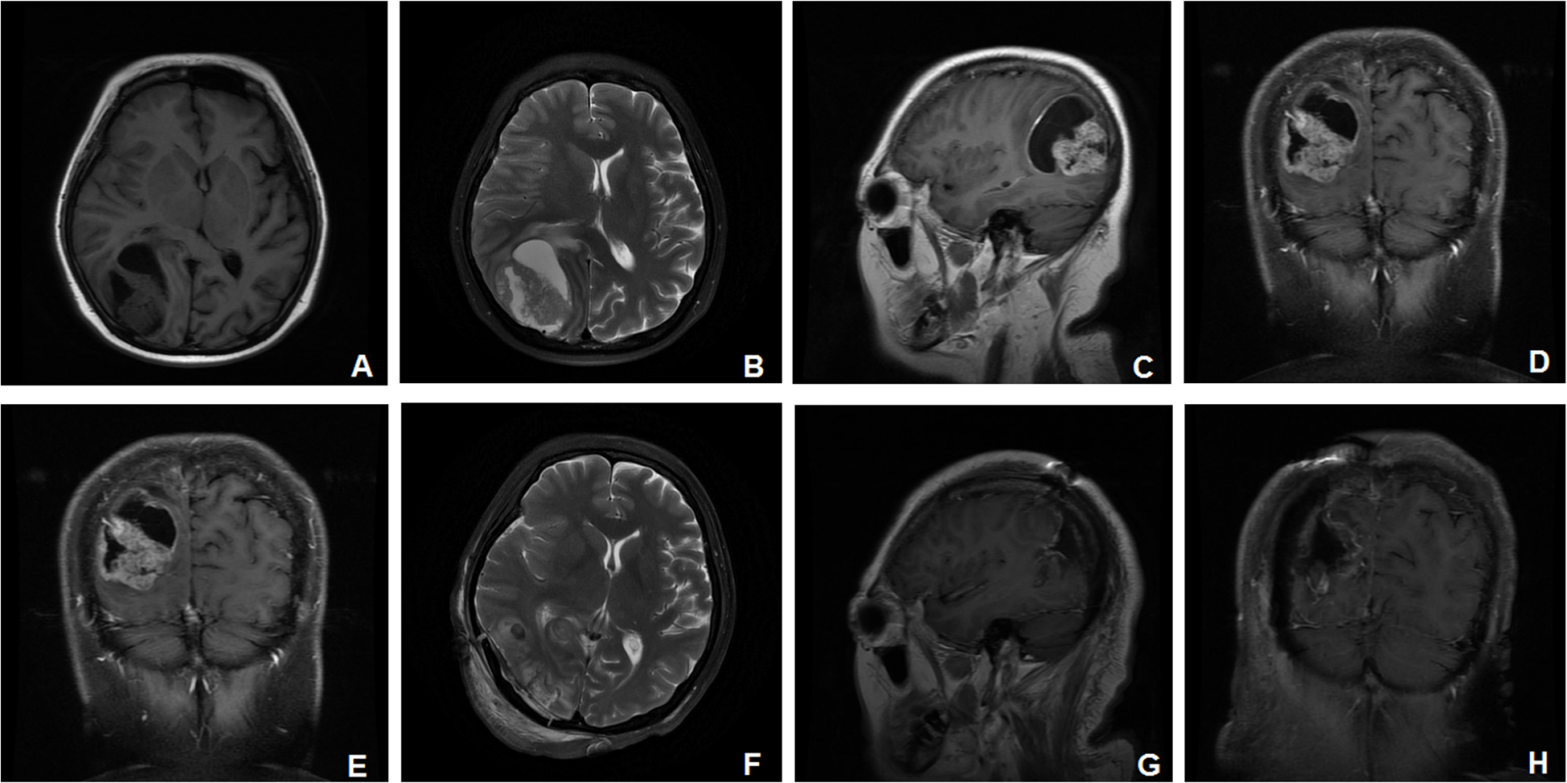
A preoperative MRI scan of the brain on July 24, 2017, showed a solitary mass in the right occipital lobe (**a**–**d**). An MRI scan of the brain 1 day after the operation showed postoperative changes in the right occipital area, with gross total resection of the lesion on August 1, 2017 (**e**–**h**). The MRI sequences consisted of the following: T1-weighted imaging (T1WI) for **a**, **g**, and **h**; T2WI for **b** and **f**; and diffusion-weighted imaging (DWI) for **c**, **d**, and **e**

**Fig. 2:**
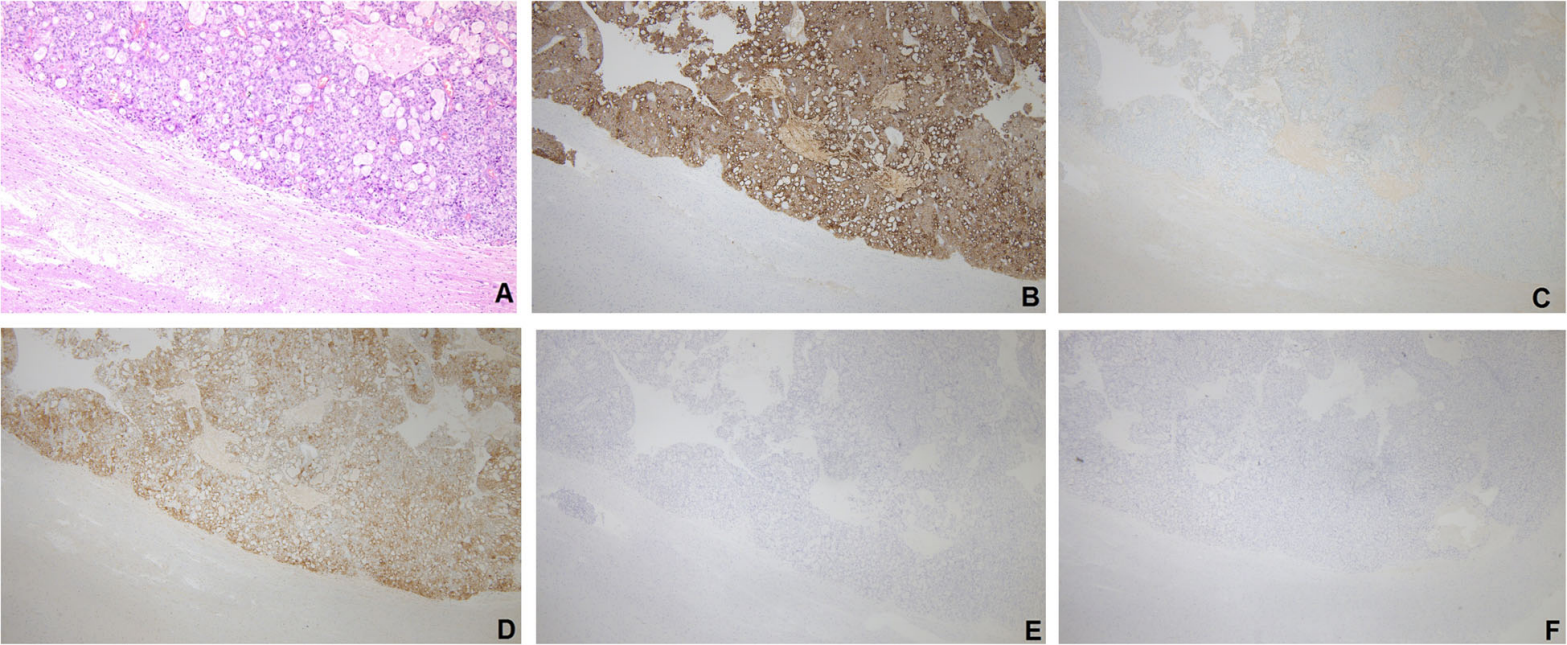
Pathology of a metastatic brain tumor from CC, which shows poorly differentiated adenocarcinoma with neuroendocrine differentiation. **a** Hematoxylin and eosin staining, × 20. Immunohistochemical staining showed strong, positive signals for CK7 (**b**), CK20 (**c**), P16 (**d**), P40 (**e**), and TTF-1 (**f**), × 20

After a 1-year remission, in September 2016, the patient presented with a cough that had lasted several weeks. CT scan of the lungs showed multiple metastatic nodules, and the biopsy revealed a poorly differentiated carcinoma. She then received 6 cycles of chemotherapy (docetaxel and cisplatin for 3 cycles, 3 weeks per cycle, and docetaxel and oxaliplatin for another 3 cycles, 3 weeks per cycle, due to decreased renal perfusion). Three months later, the patient was admitted for neurological evaluation because of severe headache, projectile vomiting, and a left homotropic hemianopia. A subsequent brain MRI showed a solitary 3 × 4 × 5 cm heterogeneous cystic mass in the right occipital lobe with surrounding edema (Fig. 1a–d). T1-weighted imaging (T1WI) and T2WI showed a solid component of equal signal, while diffusion-weighted imaging (DWI) showed hyperintensity with decreased apparent diffusion coefficient (ADC) values. The mass was enhanced with contrast enhancement. The patient underwent surgical resection of the metastatic tumor in the right occipital lobe with neuronavigation on July 31, 2017. Her preoperative Karnofsky performance score (KPS) was 80. Histopathological examination showed a metastatic, poorly differentiated adenocarcinoma with neuroendocrine differentiation (Fig. 2a). Immunohistochemical staining showed strong positivity for CK7 and positivity for CK20, P16, P40, and TTF-1 (Fig. 2b–f). The patient experienced no postoperative complications and showed good recovery at the time of discharge. A postoperative brain MRI performed on August 1, 2017, showed gross total resection of the lesion (Fig. 1e–h). Only sheet signals of long/short T1 and T2 signals were observed, and DWI still showed hyperintensity with decreased ADC values. She refused radiotherapy for rapid progression in the liver and lungs and subsequently received 5 cycles of chemotherapy (liposomal doxorubicin and carboplatin, 4 weeks per cycle) until February 2018; she died of extensive metastasis to multiple organs 2 months later in April 2018, with an overall survival (OS) of 9 months after the brain surgery.

### Case 2

A 55-year-old female underwent simple hysterectomy for assumed cervical intraepithelial neoplasia 3 (CIN 3) in July 2015. However, pathology revealed poorly differentiated squamous cell cancer of the cervix with parametrial and full-thickness stromal involvement. Immunohistochemical staining was not performed in this case. Postoperative positron emission tomography (PET) revealed multiple metastatic sites, including the lungs, mediastinum, bones, and pelvic and supraclavicular lymph nodes, confirming a diagnosis of stage IVB cancer. She was transferred to our center and was treated by external radiation to the pelvis (56.0 Gy/28 f) followed by intracavitary brachytherapy (24 Gy/4 f) and chemotherapy (cisplatin and fluorouracil, 3 weeks per cycle for 2 cycles, cisplatin and paclitaxel, 3 weeks per cycle for 6 cycles, and carboplatin and paclitaxel, 3 weeks per cycle for 1 cycle). Complete response was achieved.

**Fig. 3:**
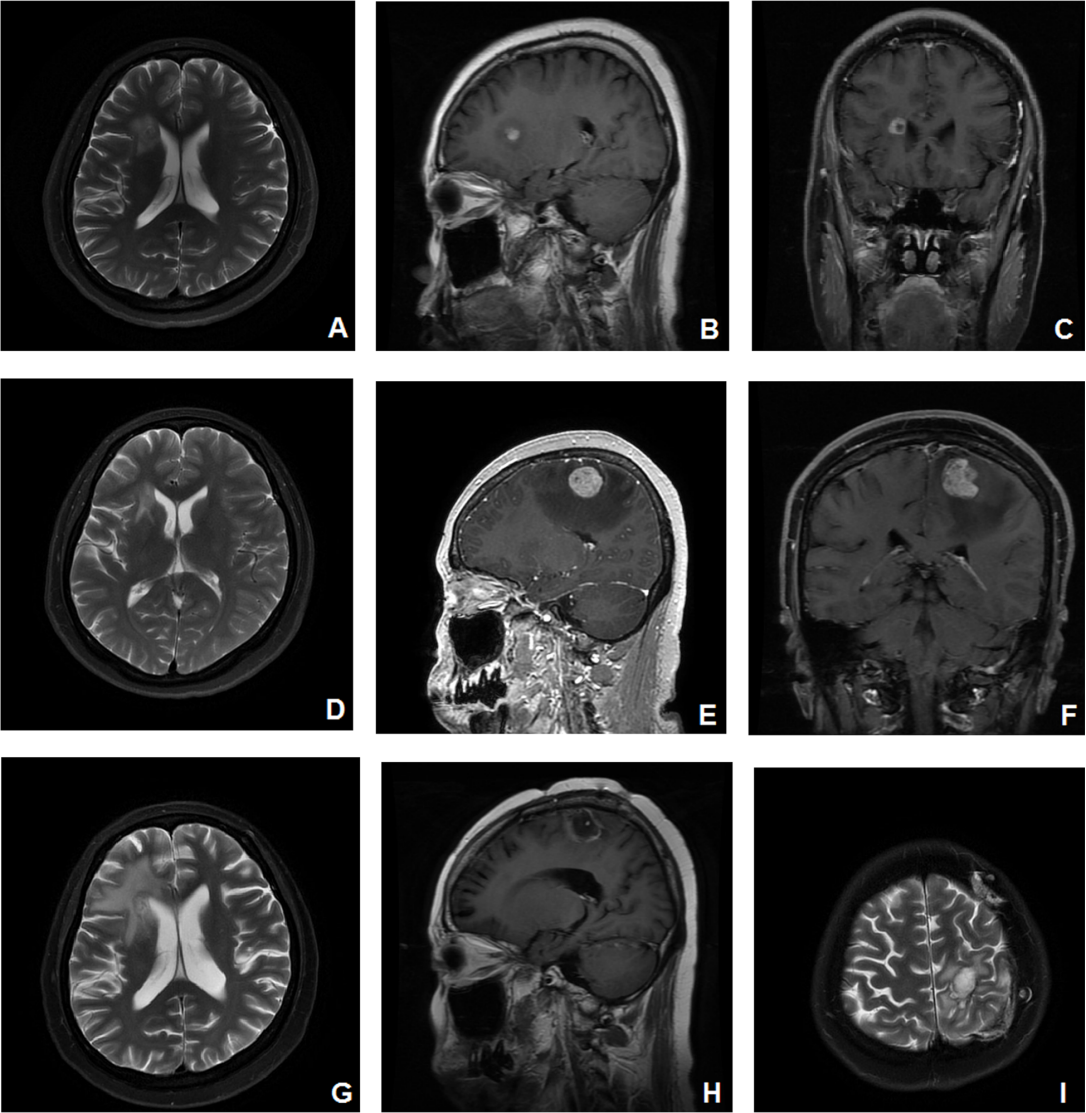
A preoperative MRI scan of the brain on November 18, 2016, showed a solitary mass in the right frontal lobe (**a**–**c**). One year later on December 25, 2017, a preoperative MRI scan of the brain showed multiple lesions in the bilateral frontal-parietal lobe (**d**–**f**). Three months after the operation, an MRI scan of the brain performed on May 3, 2018, showed postoperative changes in the bilateral frontal-parietal lobe, with gross total resection of the lesions (**g**–**i**). The MRI sequences consisted of the following: T2WI for **a**, **d**, **g**, and **i**; DWI for **b**, **c**, **e**, and **f**; and T1WI for **h**

**Fig. 4:**
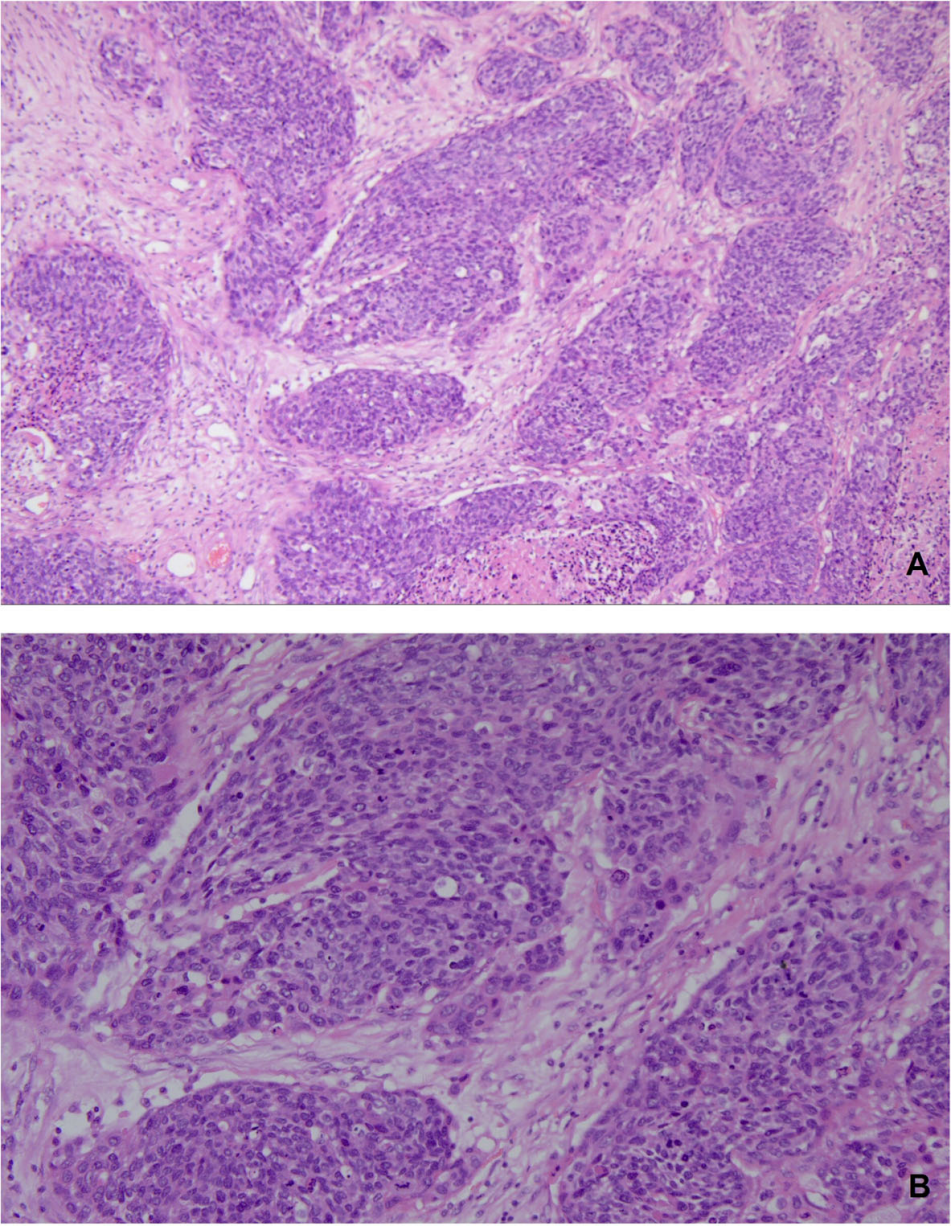
Pathology of a metastatic brain tumor from cervical squamous carcinoma (hematoxylin and eosin staining, × 20 in **a** and × 50 in **b**)

In November 2016, she presented with a mild, intermittent headache, and an MRI scan of her brain showed a solitary mass in her right frontal lobe, which could not be resected due to the location of the tumor (Fig. 3a–c). Long T1 and long T2 signals with limited DWI diffusion were observed on MRI. The mass was enhanced with contrast enhancement. Consultation with a radiotherapist led to the recommendation of whole-brain radiation therapy (WBRT) for brain metastasis. The patient refused radiotherapy for fear of both potential complications and the misdiagnosis of metastasis. During the follow-up period, the patient had a mild headache, and MRI revealed a stable lesion. In November 2017, the patient presented with weakness and trembling in her right limbs. An MRI scan of her brain showed multiple heterogeneous solid masses in the bilateral frontal-parietal lobe with surrounding edema, suggestive of brain metastases. Short T1 and long T2 signals and slightly increased DWI signals were observed, which were all enhanced (Fig. 3d–f). A CT scan of her abdomen and pelvis did not reveal any new lesions. The patient underwent surgical resection of multiple metastases in the left frontal lobe with neuronavigation on January 30, 2018. Her preoperative KPS was 70. Histopathological examination of the resected tumor revealed metastatic squamous cell cancer from the cervix (Fig. 4). The lesion in her right frontal lobe was left in situ as the patient refused surgical treatment for fear of misdiagnosis because the lesion had remained stable over the past year, even after close communication. Three months after the operation, an MRI scan of her brain showed gross total resection of the lesion, which showed long T1 and T2 signals and high DWI signals without decreased ADC values (Fig. 3g–i). The patient recovered well and showed no neurological complications postoperatively. She was subsequently treated with stereotactic radiosurgery (SRS) at a dose of 18 Gy in March 2018. She remained disease-free until December 2018 and died in March 2019 due to disease progression, with an OS of 14 months after the brain surgery.

## Discussion

Brain metastasis from CC is rare. In a review of 1565 patients diagnosed with CC, only 12 cases of brain metastasis (0.76%) were identified [10]. Pathologically confirmed brain metastasis is even rarer. Among the 149 patients with CC metastasis to the brain reported in the English literature, only 46 patients (30.9%) underwent surgical excision and had corresponding histopathological evidence [7]. These findings are contrary to those for other malignancies. Metastatic brain tumors are the most common intracranial neoplasms in adults, with an incidence of 10–15% of all patients with systemic malignancies [11, 12]. Recently, an increase in the incidence of brain metastasis has been noted, largely due to earlier detection as a result of improved imaging methods [13]. The most common primary cancers that metastasize to the brain are lung cancer (45%), breast cancer (15%), melanoma (10%), and colorectal cancer (5%) [14, 15].

These patients with brain metastasis from CC had a distinct natural history and obvious neurological symptoms. The median age of the onset of brain metastasis was 48 years, ranging from 29 to 87 years [7, 16, 17]. While some patients had brain metastasis at the time of primary cancer diagnosis [18, 19], the median interval between primary cancer diagnosis and brain metastasis was 17.2 months [7], and the longest interval reported was 127.2 months [9]. The intervals in our cases from the time of CC diagnosis to brain metastasis were 26 and 16 months. Patients suffering from brain metastasis can present with various symptoms, including headaches, seizures, cognitive impairment, fatigue, and focal deficits [20]. The most common symptoms were headache (31%), hemiplegia (16%), seizure (11%), and confusion (9%) [7]. Most patients had multiple lesions (55%), and the most common area of metastatic lesions was the supratentorial region (75%) [7].

The pathogenic mechanisms underlying brain metastasis from CC remain unclear. It has been reported that the interaction between tumor cells and brain cells within the brain microenvironment results in the release of various cytokines that subsequently promote tumor growth [21–24]. Another significant bottleneck in understanding the pathogenesis of brain metastasis is the limited availability of nutrients in the brain. Metastatic brain tissue is able to utilize substrates from glycolysis, the pentose phosphate pathway [25], and the glutamine pathway, including pyrimidines, purines, and nonessential amino acids, as both energy sources and cellular building blocks [26]. However, these findings were almost all discovered in cases of other malignancies, not in cases of CC. No substantial evidence has been uncovered to reveal the pathogenesis of brain metastasis from CC.

Among various clinicopathological factors, highly invasive subtypes or advanced stages of CC may be the key factors of metastasis to the brain. Most brain metastases from CC have been reported to be poorly differentiated [27], and nonsquamous subtypes account for 32% or more cases of brain metastasis [7, 28], which is significantly higher than the proportion in primary cervical lesions. It is worth noting that almost half of the patients (up to 40%) with intracranial metastasis from CC had advanced stage disease [7, 16]. In another report of 27 patients, 21 (77.8%) had stage IIB disease or more advanced stages of disease [29]. Most patients with brain metastasis also developed recurrence at extracranial sites, including in the lungs (39%), bones (16%), and abdomen/pelvis (16%) [7]. Small cell neuroendocrine carcinoma of the cervix is prone to brain metastasis and has an unfavorable prognosis [30]. Several reports have also documented brain metastasis in cervical neuroendocrine tumors [28, 31]. In conclusion, an advanced stage and nonsquamous subtype contribute significantly to brain metastasis, which is similar to the risk factors for bone metastasis [32, 33] or metastasis to other distant sites beyond the pelvic cavity [34]. In our two cases, the first patient had poorly differentiated stage IIB cervical carcinoma with neuroendocrine differentiation and the second patient had poorly differentiated stage IVB CC. However, more cases are needed to further uncover the high risk factors of brain metastasis from CC.

There is still no consensus on the most effective therapy for patients with brain metastasis. WBRT was suggested in the 1980s to prevent neurological death by reducing the tumor volume and treating micrometastases. WBRT is also an option for patients with uncontrolled primary disease or extensive systemic metastases; it is the treatment of choice in patients who are not suitable for surgery or SRS [35] and is used an adjuvant treatment to surgery or SRS to increase local and distant tumor control [36]. SRS employs multiple, highly focused, convergent beams to deliver a high dose of radiation to intracranial targets [37], which allows radiation to be delivered with a steep radiation dose drop-off outside the targeted tumor border, minimizing the risk of damaging the surrounding normal brain tissue [38]. SRS may be preferred to WBRT in select patients who have undergone the total resection of one to three metastatic brain lesions, as in our report of case 2. In recent studies, WBRT was associated with various short-term and long-term radiation-induced injuries to the brain. Adding stereotactic radiosurgery to WBRT provides better local control than WBRT alone, as shown in a previously reported review and meta-analysis [39]. Brown et al. [40] studied 194 patients who underwent brain metastasis resection and found no difference in survival between SRS and WBRT, while cognitive impairment was more frequent in patients who received WBRT than in those who received SRS at 6 months (85% vs. 52% of patients, *p* < 0.001). Novel therapies for brain metastasis from malignancies are emerging, including targeted therapy. These reports include cases of melanoma brain metastasis treated with ipilimumab [41, 42] and cases of brain metastasis from melanoma and non-small-cell lung cancer treated with pembrolizumab [43]. To the best of our knowledge, there are no targeted therapies for brain metastasis from CC.

Surgical resection is now considered for patients with a radioresistant primary histological type, those with a large tumor volume causing brain shift, those with symptoms refractory to medical treatment, and those with controlled disease at the primary site without systemic metastasis [13]. However, very little of this experience is in the field of CC. A small cohort study revealed that patients who underwent surgery for brain metastasis exhibited better survival than patients receiving only WBRT [44]. Favorable prognostic factors for prolonged survival after the surgical resection of central nervous system metastases are a good patient performance status, a long disease-free interval, an absence of other systemic diseases, and resectability, preferably with clear margins [45]. Additionally, resection allows for the histological confirmation of metastasis and differentiation with necrosis [46, 47]. It is alarming that this evidence was not all drawn from the treatment of CC but was instead drawn from the treatment of heterogeneous tumor types. Thus, the adoption and generalization of these conclusions in CC patients should be considered with caution. The surgical timing for brain metastasis has not been explicitly clarified. Surgical treatment in our two cases achieved transient disease-free periods of 7 and 11 months, demonstrating the positive role of surgery. Interestingly, in case 2, a single right frontal lobe lesion was stable for 1 year before new lesions appeared in the bilateral frontal-parietal lobe.

The survival of brain metastasis from CC is very poor; the mean and median survival times after the diagnosis of brain metastasis were reported to be 7 and 4.6 months, respectively, in a literature review [7]. Records from 81 patients with uterine cancer metastasis to the brain in Japan showed a median OS of 7 months (95% CI 4–10) [48]. In another study, the mean survival was 8.2 months after central nervous system metastasis was discovered [29]. It has been suggested that the treatment modality, particularly combined therapies, is significantly related to OS [49]. The poor prognosis is probably due to metastasis to multiple sites rather than to the brain alone. In our cases, after brain surgery and multimodal therapy, the OS in cases 1 and 2 was 9 and 14 months, respectively. Both patients died of disease progression, even after successful management of the brain loci.

## Conclusion

Pathologically confirmed brain metastasis from CC is rare. Although management varies based on individual characteristics, surgery appears to be critical for both disease control and pathological confirmation. Highly invasive subtypes or advanced stages of CC may be the key factors of brain metastasis. Future large-scale reports are needed to clarify the pathogenesis of and optimal treatment approach for brain metastasis from CC.

## Data Availability

All the data in this report have been presented in the manuscript.
